# Relationship between Reaction Times and Post-COVID-19 Symptoms Assessed by a Web-Based Visual Detection Task

**DOI:** 10.3390/healthcare11030284

**Published:** 2023-01-17

**Authors:** Natale Vincenzo Maiorana, Edoardo Nicolò Aiello, Barbara Poletti, Fabrizio Carusi, Angelica De Sandi, Matteo Guidetti, Roberto Prandin, Sara Marceglia, Nicola Ticozzi, Vincenzo Silani, Alberto Priori, Roberta Ferrucci

**Affiliations:** 1 Aldo Ravelli Center for Neurotechnology and Experimental Brain Therapeutics, Department of Health Sciences, International Medical School, University of Milan, 20142 Milano, Italy; 2 Department of Neurology and Laboratory of Neuroscience, IRCCS Istituto Auxologico Italiano, 20149 Milan, Italy; 3 ASST Santi Paolo e Carlo, San Paolo University Hospital, 20142 Milano, Italy; 4 Department of Electronics, Information and Bioengineering, Politecnico di Milano, 20133 Milan, Italy; 5 Department of Engineering and Architecture, University of Trieste, 34127 Trieste, Italy; 6 Department of Pathophysiology and Transplantation, Dino Ferrari Center, University of Milan, 20122 Milan, Italy

**Keywords:** COVID-19, SARS-CoV-2, neuropsychology, attention, post-COVID syndrome

## Abstract

Long-COVID is a clinical condition in which patients affected by SARS-CoV-2 usually report a wide range of physical and cognitive symptoms from 3 to 6 months after the infection recovery. The aim of the current study was to assess the link between self-reported long-COVID symptoms and reaction times (RTs) in a self-administered Visual Detection Task (VDT) in order to identify the predictor symptoms of the slowing in reaction times to determine attention impairment. In total, 362 participants (age (mean ± S.D.: 38.56 ± 13.14); sex (female–male: 73.76–26.24%)) responded to a web-based self-report questionnaire consisting of four sections: demographics, disease-related characteristics, and medical history questions. The final section consisted of a 23 item 5-point Likert-scale questionnaire related to long-term COVID-19 symptoms. After completing the questionnaire, subjects performed a VDT on a tablet screen to assess reaction times (RTs). An exploratory factorial analysis (EFA) was performed on the 23 long-COVID symptom questions, identifying 4 factors (cognition, behavior, physical condition, presence of anosmia and/or ageusia). The most important predictors of RTs were cognition and physical factors. By dissecting the cognitive and physical factors, learning, visual impairment, and headache were the top predictors of subjects’ performance in the VDT. Long-COVID subjects showed higher RTs in the VDT after a considerable time post-disease, suggesting the presence of an attention deficit disorder. Attention impairment due to COVID-19 can be due to the presence of headaches, visual impairments, and the presence of cognitive problems related to the difficulty in learning new activities. The link between the slowing of reaction times and physical and cognitive symptoms post-COVID-19 suggests that attention deficit disorder is caused by a complex interaction between physical and cognitive symptoms. In addition, the study provides evidence that RTs in a VDT represent a reliable measure to detect the presence of long-COVID neurological sequelae.

## 1. Introduction

SARS-CoV-2 is a human coronavirus that causes the respiratory infectious disease known as COVID-19 and, due to its high contagiousness, caused a global pandemic in the first months of the 2020s that is still ongoing [1,2]. In its worst form, SARS-CoV-2 infection causes severe lung damage and breathing disorders that require hospitalization in intensive care units (ICUs), representing a burden on healthcare systems [3]. A large number of patients report an expanded spectrum of physical, psychiatric, neurological, and neuropsychological dysfunctions over the course of the disease and/or up to 3 to 6 months after recovery [4,5,6,7,8,9]. This condition has been named “long-COVID”, or “post-COVID-19 syndrome” by the worldwide scientific and medical communities [10]. The consequences of SARS-CoV-2 infection appear to affect both hospitalized and non-hospitalized patients [11]. Due to the complexity of COVID-19 clinical manifestations, it was difficult to develop a defined clinical pathway for the management of COVID-19 patients [6,12]. Neurological symptoms such as cognitive impairment during COVID-19 infection and post-COVID-19 syndrome could be due to hypoxia occurring during infection, SARS-CoV-2 mediated neuroinflammation, or the presence of SARS-CoV-2 in the brain tissue [13,14]. To date, it is still unclear whether the neurological consequences of COVID-19 are due to hypoxic brain tissue deprivation of oxygen or to a direct effect of the virus on the brain [15,16,17,18,19].

Post-COVID-19 patients have been found to exhibit reduced gray matter thickness in the orbitofrontal cortex and parahippocampal region [20]. Regardless of their cause, long-term cognitive changes due to COVID-19 infection are commonly reported, such as impairments in memory, concentration, attention, and executive functioning. In a study of a US sample of 5163 respondents, it was found that 50% of respondents reported difficulty focusing and concentrating [21]. In addition, dysfunctions of executive functions and attention were found [22]. Similar results were found in a large sample composed of 84,285 respondents with a suspected or confirmed COVID-19 infection diagnosis that presented with cognitive impairment related to memory, attention, and executive functioning [23].

To date, the association between cognitive impairment and other clinical symptoms due to COVID-19 infection remains unclear, with conflicting evidence pointing to the role of hospitalization or the presence of specific symptoms during the illness [18]. However, the most common symptoms, such as fatigue and cognitive impairment, were found in both hospitalized and non-hospitalized patients who had recovered from SARS-CoV-2 infection [23,24].

The persistence of fatigue and cognitive impairment was indicated by self-reports and subjective measures (via online/electronic questionnaire, systematic telephone interview or email survey) [25,26,27,28] and objective assessment, also using various tests, such as the Modified Telephone Interview for Cognitive Status (TICS-M) [29], the Cognitive Failures Questionnaire (CFQ), the Screen for Cognitive Impairment in Psychiatry Danish Version (SCIP-D) and the Trail Making Test-Part B (TMT-B) [30,31], the Mini-Mental State Examination [32,33], the Brief Assessment of Cognition in Schizophrenia (BACS) [34], and the Montreal Cognitive Assessment (MoCA) [5,18,35,36]. The main deficits associated with cognitive impairment were difficulty concentrating, long-term verbal and spatial memory impairment, forgetfulness, and thinking difficulties. In addition, these dysfunctions resulted in problems in patients’ quality of life, as assessed using the European Quality of Life Scale 5 Dimensions 5 Levels (EQ-5D-5L) or EQ-5D-3L scale to examine their functional outcomes known as quality of life (QoL) measures [24].

Post-COVID-19 patients experience both cognitive and physical symptoms. The copresence of symptoms such as attention, executive functions, mental, and physical fatigue could be due to shared neural networks involved in the subjective experience of cognitive and physical fatigue [37].

Furthermore, mental fatigue has an impact on psychophysiological responses leading to poor cognitive performances [38]. From a clinical standpoint, it was demonstrated that a combined physical and cognitive rehabilitation program was more effective in increasing cognitive performance, compared to training involving only physical or cognitive training [39].

Patients who recovered from COVID-19 had a decrease in quality of life, reporting pain or discomfort, problems with self-care and mobility, and health-related impairments at work and during activities. Some patients did not return to work or resume sports after hospitalization [30,31,40,41,42]. Large-scale cognitive assessment of post-COVID-19 patients may shed light on the relationship between experienced COVID-19 symptoms and cognitive impairment [5,43]. So far, the screening of post-COVID-19 patients can be carried out with online applications and questionnaires; the use of information technology enables large-scale screening and collection of data on patients who cannot be tested due to their clinical condition or distance from clinical structures.

In recent years, the use of mobile-based cognitive assessments has increased, particularly in the areas of memory and attention through the management of reaction times [44].

To date, it is of interest to better understand the link between physical and cognitive symptoms and their relationship with cognitive performance on neuropsychological tests. Understanding the relationship between long-COVID experienced symptoms and cognitive functioning could provide useful insight for clinicians in daily practices.

In the current study, we present the results of a web-based questionnaire examining the main symptoms in post-COVID-19 patients, the main symptoms experienced during COVID-19 infection, and performance on a reaction time task assessing attention deficit in post-COVID-19 patients. The aim of the present study was to assess the relationship between self-reported symptoms associated with post-COVID-19 syndrome and reaction times to determine which are associated with slower reaction times.

## 2. Methods

### 2.1. Participants

A web-based questionnaire, followed by a single stimulus VDT, was shared via an online form, webmail, and social media, and it was completed by N = 376 post-COVID-19 native Italian speakers as subjects who consented to participate.

The study was conducted between March and July 2022 and included native Italian speakers, both male and female, in the age range between 18 and 65 years old. The convenience sample of participants was reached through social media and instant messaging platforms. As we aimed to reach the general population, the only inclusion criterion was the age of majority (>18 years).

People were invited to participate in the study via social media and email. The procedure involved filling out an online consent form. All data were collected anonymously and stored in a password-protected electronic format. The study was approved by the Institutional Ethics Committee of the San Paolo Hospital of Milan (Reg. n. 2020/ST/105) and was conducted according to the guidelines of the Declaration of Helsinki. There was 1 subject excluded due to unreliable responses, while N = 14 due to co-morbid brain disorders, i.e., Parkinson’s disease (N = 4), multiple sclerosis (N = 4), epilepsy (N = 2), Hashimoto’s disease (N = 3), and panic attack disorder (N = 1). The remaining subjects (N = 361) were free of (1) neurological/psychiatric diagnoses, (2) uncompensated general-medical conditions, and (3) uncorrected sensory deficits. Subjects’ clinical features are reported in Table 1, and demographic data of the sample are reported in Table 2.

### 2.2. Materials

The questionnaire covered the following areas: (1) demographics (i.e., age, gender, education, regional origin); (2) disease-related characteristics (i.e., month and year of infection(s), disease severity); (3) medical history (i.e., relevant morbidities, active medications); (4) a validated 23-item 5-point Likert-scale questionnaire on the severity of a set of neurological, neuropsychological, and physical signs/symptoms (where 0 corresponds to the absence of a specific sign/symptom and 4 to maximum severity) [45]. The administered questionnaire was representative of the full spectrum of neurological sequelae of COVID-19. The symptoms and signs considered in this assessment were chosen according to the existing literature on post-COVID-19 syndrome [41,42] and were agreed on by a panel of neurologists and neuropsychologists with expertise in post-COVID-19 neurological outcomes to be appropriate for the above objective. Table 3 shows the questionnaire. A VDT followed the questionnaire and asked participants to tap as quickly as possible where a red dot appeared on the phone screen. Ten attempts were made, the first being considered a catch attempt; the result of the VDT was thus represented by the means of the following nine trials.

### 2.3. Statistical Analysis

Sample size was estimated according to the previous literature that proposes a minimum sample size of 100 subjects for exploratory factorial analysis [46] and between 20 and 80 for regression models of health measurement in neurology [47].

A data-driven dimensionality-reduction approach was embraced with the aim of identifying a parsimonious set of potential predictors featured by optimal measurement properties. First, an exploratory factor analysis (EFA) was performed on the 23-item questionnaire by adopting a parallel analysis-based, Maximum Likelihood Extraction Method, and an oblimin rotation. A simple structure of 4 correlated (*r* ≥ 0.2) factors was detected, with only two items (i.e., “Hearing disturbances” and “Hypersensitivity to environmental stimuli”) being featured by a primary loading < 0.35. After removing such items, the four-factor structure remained unchanged and improved in simplicity (i.e., all primary loading being > 0.35; range = 0.36–0.98; 58.3% of variance cumulatively explained). Items loading on each factor are displayed in Table 4. Briefly, the four factors represented are *cognition*, *behavior*, *physical status,* and *anosmia and ageusia*. Internal reliability, as tested via Cronbach’s α, was optimal for each factor (*cognition*: 0.94; *behavior*: 0.88; *physical status*: 0.75; *anosmia and ageusia*: 0.91).

Thereupon, factorial scores for each subject were computed via Anderson and Rubin’s (1956) [48] approach and entered into a multiple regression model along with age, sex, education (ordinal scale), time from disease onset (days), self-rated disease severity (ordinal scale), and the occurrence of multiple infections (“yes” vs. “no”). Since the mean RTs in the VDT showed a clear floor-effect and a high inter-individual variability (i.e., skewness and kurtosis values > |1| and |3|, respectively) [49], a generalized linear model underlying a Gamma distribution and addressing a logarithmic link function was employed. Indeed, Gamma distributions allow the modeling of right-skewed, over-dispersed continuous data [50]. Within the Gamma regression model, a backward elimination approach was adopted, consisting in entering all the above-mentioned predictors simultaneously and then dropping those with the highest non-significant *p*-values at the time. The significance threshold considered for retaining predictors was set at α = 0.05. If one factor happened to yield significance within the aforementioned model, a further Gamma regression was run in order to determine which specific items contribute to its effect. In such cases, the same backward elimination process was addressed. Analyses were run in jamovi 2.3.12 (https://www.jamovi.org/ (accessed on 1 December 2022)) and R 4.1.0 (https://www.r-project.org/ (accessed on 1 December 2022)).

## 3. Results

**Figure 1:**
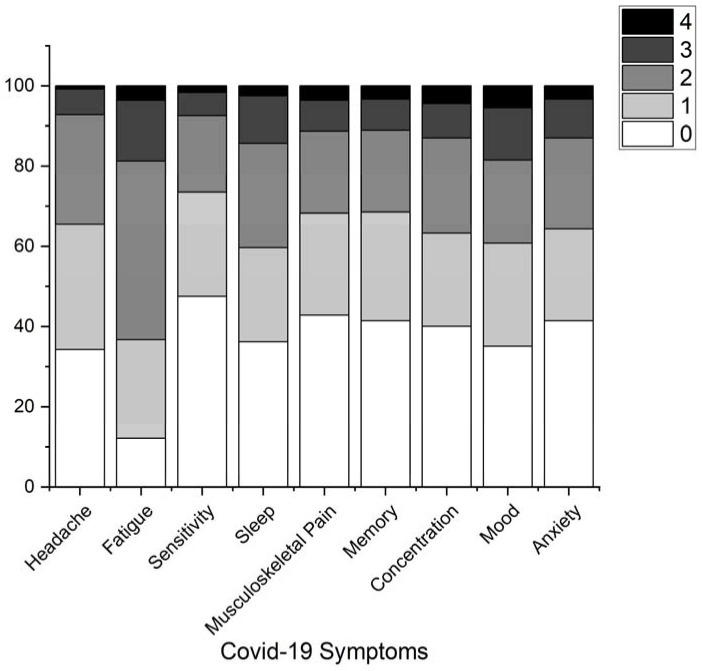
Stitch bar graph representing symptoms experienced by at least 45% of the sample. Symptom severity grouped as in the questionnaire responses: 0 = none; 1 = slight; 2 = mild; 3 = moderate; 4 = severe.

From a descriptive point of view, the most commonly experienced symptoms in our sample were fatigue (87.84%), headache (65.74%), mood disorders (64.91%), sleep disorders (63.81%), concentration and attention problems (59.94%), anxiety (58.56%), memory impairment (58.56%), musculoskeletal pain (57.18%), and sensitivity to external auditory stimuli (52.48%) (Figure 1). Table 5 summarizes subject responses to the questionnaire and the VDT. Within the baseline level of Gamma regression, no predictor yielded significance *(p* = 0.062) except for age (χ^2^(1) = 45.93; *p* < 0.001) and physical status (χ^2^(1) = 5.47; *p* = 0.019). In the subsequent steps, education (*p* = 0.531), occurrence of multiple infections (*p* = 0.415), gender (*p* = 0.41), behavior (*p* = 0.372), anosmia and ageusia (*p* = 0.34), severity of the disease (*p* = 0.104), and disease duration (*p* = 0.175) were removed in that order. The final model then included age (χ^2^(1) = 41.6; *p* < 0.001), *cognition* (χ^2^(1) = 6.96; *p* = 0.008), and *physical status* (2(1) = 6.02; *p* = 0.014) which positively predicted all RTs in the VDT (i.e., poorer cognitive and physical statuses predicted higher RTs). When decomposing the effect of *cognition* by also including age (which stayed significant: *p* < 0.001), the backward elimination process yielded only the *learning* item as a positive predictor of RTs (χ^2^(1) = 12.9; *p* < 0.001; i.e., worse learning abilities predicted higher RTs). For the decomposition of the effect of physical condition, again adjusting for age (*p* < 0.001), only higher scores (corresponding to more severe signs/symptoms) on the items visual disturbances and headache predicted higher RTs (*visual disturbances*: χ^2^ (1) = 4.58; *p* = 0.023; *headache*: χ^2^ (1) = 8.1; *p* = 0.004).

## 4. Discussion

The aim of the present study was to assess attentional functions of post-COVID-19 participants using a web-based VDT and to clarify the relationship between attention deficits (i.e., slowing of RTs) and the main symptoms that appear after the onset and progression of the disease. To the best of our knowledge, this is the first study to perform a web-based, self-administered cognitive assessment in an Italian sample of post-COVID-19 subjects. First, we performed a factorial analysis of the questionnaire responses to identify the factors that could represent the main symptoms that participants experienced in the months following recovery from COVID-19.

The factorial analysis of the responses to the self-report questionnaire showed that post-COVID-19 symptoms can be divided into four domains: cognition, behavior, physical status, and the presence of anosmia and ageusia. As other studies have noted, post-COVID-19 syndrome is characterized by multiple symptoms such as fatigue, dizziness, attention, memory, and executive function problems that affect all daily activities [5,30,44]. The specific causes of this disease are still unknown. Post-COVID-19 syndrome could be due to a hypoxic state that occurred during the course of infection [5,10,40], where oxygen starvation could lead to brain tissue degeneration or to neuroinflammation due to the presence of SARS-CoV-2 in brain tissue [51,52,53,54].

The most important structures that might be involved in tissue degeneration are the hippocampal region and the prefrontal cortices [52]. Some studies drew their attention to a condition called happy hypoxia [52,55,56,57]. With happy hypoxia, patients show low blood oxygen saturation, accompanied by symptoms in the lower respiratory tract [55]. In this condition, however, low levels of oxygen in the blood can affect brain structures and lead to degeneration. Another cause of cognitive impairment could be the presence of SARS-CoV-2 in brain tissue. The presence of ageusia and anosmia may reflect the existence of SARS-CoV-2 in the prefrontal cortices [58], specifically in the primary olfactory cortex, a region functionally associated with the parahippocampal and orbitofrontal cortices [58,59,60].

Considering the burden of cognitive impairment on all daily activities and on social costs, it is of interest to find out the main factors associated with cognitive impairment in post-COVID-19 syndrome [61]. Clarifying the relationship between experienced symptoms and cognitive impairment could provide useful insights for clinicians in the diagnosis and rehabilitation of this category of patients [62,63].

We found that physical and cognitive symptom factors predicted RTs in the VDT. Specifically, by dissecting physical and cognitive factors, we found that learning impairment (cognitive factor), visual impairment, and headache (physical factor) were the most important predictors of slowed RTs.

Previous studies found that SARS-CoV-2 infection can affect the visual system [62,63]. Patients who have recovered from COVID-19 are at higher risk of developing changes in the visual cortex and optic nerve [62,63,64]; as a consequence, they might experience vision loss and visual impairment [65]. Slowing in RTs could be due to a visual impairment that reduces the ability to recognize stimuli.

The other point related to the physical condition that explains the slowing in reaction times was the presence of a headache. Headaches and migraines are a well-known consequence of COVID-19 and one of the main symptoms of long-COVID [66]. Most participants in our study reported headaches, ranging in severity from mild to severe. A study conducted by Attridge et al. [67] found that headaches can increase RTs on complex attentional tasks and that such a slowing may be due to the involvement of fundamental attentional processes, as demonstrated by subjects’ performance on a selected RT task. Headaches appear to affect the basic cognitive components necessary to perform attentional tasks, regardless of task complexity [68].

In addition, we found that the cognition factor predicted RTs in the VDT. This result indicates that patients reporting symptoms in the cognitive domain had slower RTs in the VDT. Reaction times are a valid measure for determining cognitive impairment [69,70,71]. Symptoms experienced in the cognitive domain could be due to a deficit in information processing and attention remarked by the slowing in reaction times. Even if cognition is composed of complex interactions between brain functions, a psychomotor slowing in reaction times denotes a difficulty which could predict a more complex cognitive impairment [71,72]. By decomposing the effect of the cognition factor, we found a significant effect of the learning impairment as a predictor of reaction times in the VDT. The learning item denotes deterioration in the ability to learn novel information or the ability to perform unprecedented and unfamiliar tasks. It may be possible that the slowing of RTs in the VDT reflects the same attention deficits that underlie the difficulty in learning new information or completing a new, unfamiliar task.

Our study has some limitations; our findings are based only on self-report by patients, without the possibility to compare the participants’ responses with their clinical records. Since we collected data only in a VDT, it would have been interesting to administer a more complex task in order to assess complex cognitive functions. Although the sample size was appropriate for EFA and the regression model on the administered questionnaire, a larger sample of participants is needed to expand the results to the general population also considering the complexity of the clinical manifestations of COVID-19.

Despite the limitations, our results are consistent with previous findings and show that post-COVID-19 syndrome is characterized by both physical and cognitive impairments [5,11,35,73]. Furthermore, our results may be useful in the comprehension of the specific cognitive domain affected in post-COVID syndrome and help to clarify the relationship between physical and neuropsychological symptoms to better define the clinical management of patients that have recovered from SARS-CoV-2 infection.

In addition, our data show that self-reported cognitive and physical impairments predict performance in a VDT in terms of slowing RTs. These results suggest that RTs recorded by an online-managed VDT could be an effective way to study post-COVID-19 syndrome from a neuropsychological perspective.

## Figures and Tables

**Table 1:** Clinical Data of the sample.

Clinical Data					
Days from Infection (Mean)		240	524	262	207
Reinfection (yes/no) %		5.5	94.5		
		Asymptomatic	Hospitalization and Oxygen Therapy	**Hospitalization and Pharmacologic Therapy**	**Flu-like Symptoms**
Severity	n	35	7	7	312
	%	9.70	1.94	1.94	86.70
		Yes	No		
Comorbidity		24.4	75.6		
Pharmacotherapy needs		27.1	72.9		

**Table 2:** Demographic Data of the sample.

Demographics				
N	361			
Age (years ± S.D.)	38.56 ± 13.14			
Age (Range)	(18–83)			
Sex (Female %/Male %)	73.76	26.24		
Education (years)	8	13	18	>18
n	16	111	118	116
Geographical Area	North	Centre	South	Islands
%	64.64	11.60	20.17	3.59

**Table 3:** Items included in the administered questionnaire. Item of the administered questionnaire. The “symptom” column shows the symptom measured by the related item.

	Self-Reported Questionnaire on Experienced Symptoms	
n.	Item	Symptom
(1)	Do you suffer from headaches?	Headache
(2)	Do you get tired easily?	Fatigue
(3)	Do you suffer from dizziness, vertigo, have problems with balance or coordination of movements?	Movement
(4)	Has your vision become blurred, or do you have moments when you seem to ‘see double’?	Vision
(5)	Do you have hearing problems?	Hearing
(6)	Does noise, light or crowded places bother you?	Sensitivity
(7)	Have you sleep problem or do you need more sleep during the day?	Sleep
(8)	Has your appetite changed (increased or decreased)?	Eating
(9)	Have you noticed any changes in taste?	Ageusia
(10)	Have you noticed any changes in your sense of smell?	Anosmia
(11)	Do you have any problems with nausea/vomiting?	Gastrointestinal
(12)	Have you experienced any blackouts or fainting?	Fainting
(13)	Have any pains, contractures or muscle weaknesses appeared?	Musculoskeletal Pain
(14)	Do you have the impression that you have become less tolerant to the alcohol effects?	Alcohol
(15)	Have you noticed memory problems (forgetting things to do, appointments, etc.)?	Memory
(16)	Have you noticed difficulties in learning new information, skills or task?	Learning
(17)	Do you find it harder to concentrate and maintain focus on a specific task?	Concentration
(18)	Do you need more time to write documents or read?	Time for information processing
(19)	Do you find yourself unable to say a word that is on the ‘tip of your tongue’?	Tip of the Tongue (TOT)
(20)	Do you have difficulty doing mental arithmetic?	Counting skills
(21)	Do you feel downhearted, frustrated, in a bad mood?	Mood
(22)	Do family/friends/colleagues tell you that you have become irritable, intolerable, or lose control easily?	Irritability
(23)	Do you get worried or agitated even by trivial events?	Anxiety

**Table 4:** Loading matrix of the extracted factors. Factors were extracted with the Maximum Likelihood Extraction Method in combination with “oblimin” rotation.

	Factor				
	Cognition	Behavior	Physical	Anosmia/Ageusia	Uniqueness
Learning	0.976				0.1724
Concentration	0.855				0.1514
Time for information processing	0.827				0.1830
Memory	0.770				0.3347
Counting skills	0.746				0.3448
TOT	0.653				0.5221
Mood		0.847			0.2587
Anxiety		0.726			0.4189
Irritability		0.691			0.3954
Fatigue		0.510			0.4681
Sleep		0.475			0.4791
Eating		0.441			0.5985
Movement			0.728		0.3963
Vision			0.586		0.4917
Fainting			0.526		0.7547
Musculoskeletal Pain			0.477		0.5256
Gastrointestinal			0.394		0.7706
Headache			0.361		0.7776
Anosmia				0.965	0.0898
Ageusia				0.846	0.2163

**Table 5:** Mean Reaction Times calculated within Symptom Severity levels for each symptom considered.

	Symptom Severity									
	None		Slight		Mild		Moderate		Severe	
Symptom	Mean	Standard Deviation	Mean	Standard Deviation	Mean	Standard Deviation	Mean	Standard Deviation	Mean	Standard Deviation
Ageusia	0.616	0.541	0.585	0.214	0.775	1.064	1.031	0.980	0.563	0.136
Alcohol	0.620	0.541	0.622	0.311	0.842	1.109	0.545	0.175	0.547	0.059
Anosmia	0.614	0.535	0.596	0.250	0.770	1.042	0.985	0.945	0.561	0.161
Anxiety	0.583	0.312	0.728	0.861	0.658	0.728	0.659	0.596	0.702	0.481
Concentration	0.607	0.649	0.661	0.527	0.700	0.736	0.670	0.342	0.546	0.125
Counting	0.562	0.278	0.735	0.907	0.853	1.044	0.738	0.441	0.559	0.117
Eating	0.608	0.588	0.595	0.274	0.789	0.893	0.705	0.301	0.601	0.113
Fatigue	0.514	0.141	0.677	0.836	0.595	0.295	0.791	0.906	0.857	0.931
Gastro-Intestinal	0.644	0.649	0.599	0.246	0.637	0.286	0.544	0.040	2.214	2.438
Headache	0.631	0.692	0.600	0.365	0.574	0.213	1.250	1.455	0.536	0.086
Hearing	0.604	0.531	0.658	0.386	0.891	1.191	1.114	1.165	0.510	0.028
Irritability	0.626	0.587	0.592	0.280	0.764	0.914	0.688	0.766	0.707	0.529
Learning	0.565	0.292	0.698	0.827	0.758	0.873	0.796	0.749	0.578	0.120
Memory	0.599	0.576	0.688	0.796	0.641	0.317	0.760	0.713	0.608	0.314
Mood	0.629	0.744	0.612	0.322	0.643	0.390	0.708	0.924	0.743	0.435
More Time for Daily Activities	0.583	0.593	0.668	0.397	0.672	0.529	0.895	1.089	0.527	0.150
Movement	0.614	0.560	0.621	0.371	0.789	0.965	0.613	0.143	1.354	1.727
Musculoskeletal Pain	0.601	0.639	0.658	0.688	0.642	0.355	0.838	0.783	0.657	0.306
Sensitivity	0.633	0.633	0.639	0.709	0.606	0.201	0.847	0.795	0.785	0.686
Sleep	0.620	0.686	0.598	0.342	0.613	0.306	0.903	1.102	0.529	0.055
Fainting	0.637	0.614	0.667	0.358	0.530	0.126	1.364	1.716		
Tip of the Tongue	0.546	0.232	0.673	0.770	0.745	0.845	0.746	0.411	0.538	0.088
Vision	0.610	0.565	0.569	0.222	0.689	0.387	1.561	2.093	0.996	0.793

## Data Availability

Datasets associated with the present study are available upon reasonable request of interested researchers.
